# Lipid Metabolism, Apoptosis and Cancer Therapy

**DOI:** 10.3390/ijms16010924

**Published:** 2015-01-02

**Authors:** Chunfa Huang, Carl Freter

**Affiliations:** Division of Hematology/Oncology, Department of Internal Medicine, School of Medicine and Cancer Center, Saint Louis University, 3655 Vista Avenue, Saint Louis, MO 63110, USA; E-Mail: freterc@slu.edu

**Keywords:** lipid metabolism, apoptosis, cancer therapy, bioactive lipid molecule, cell signaling

## Abstract

Lipid metabolism is regulated by multiple signaling pathways, and generates a variety of bioactive lipid molecules. These bioactive lipid molecules known as signaling molecules, such as fatty acid, eicosanoids, diacylglycerol, phosphatidic acid, lysophophatidic acid, ceramide, sphingosine, sphingosine-1-phosphate, phosphatidylinositol-3 phosphate, and cholesterol, are involved in the activation or regulation of different signaling pathways. Lipid metabolism participates in the regulation of many cellular processes such as cell growth, proliferation, differentiation, survival, apoptosis, inflammation, motility, membrane homeostasis, chemotherapy response, and drug resistance. Bioactive lipid molecules promote apoptosis via the intrinsic pathway by modulating mitochondrial membrane permeability and activating different enzymes including caspases. In this review, we discuss recent data in the fields of lipid metabolism, lipid-mediated apoptosis, and cancer therapy. In conclusion, understanding the underlying molecular mechanism of lipid metabolism and the function of different lipid molecules could provide the basis for cancer cell death rationale, discover novel and potential targets, and develop new anticancer drugs for cancer therapy.

## 1. Introduction

Lipids are hydrophobic or amphipathic (hydrophilic and lipophilic) small molecules, and are not like proteins, nucleic acids, and polysaccharides which are large macromolecular polymers formed by the chemical linking of several small constituent molecules (these molecular building blocks are similar, or homologous, in structure) [1,2]. Thus far, there is still no widely accepted definition of lipids because lipids comprise an enormous number of chemically distinct molecules and are structurally quite diverse. Lipids are frequently defined as naturally occurring compounds that are insoluble in water but soluble in nonpolar solvents [1,2]. Amphipathic lipids form plasma membranes in which cells can maintain all biological events in an intracellular environment and respond to the changes of extracellular environment.

In all living cells, lipids are required to maintain cellular structure, provide energy and are involved in cell signaling. Lipid metabolism (anabolism and catabolism) generates a variety of biological intermediators. Many of these intermediators are bioactive lipid molecules (also known as signaling molecules or second messengers) which are produced by the activation of multiple signaling pathways and can also regulate multiple signaling pathways [3]. Lipid metabolism connects to signaling networks in the regulation of cell growth, proliferation, differentiation, survival, apoptosis, inflammation, motility, and membrane homeostasis [4,5,6]. Meanwhile, lipid metabolism can alter membrane composition and permeability which cause the development and progression of many diseases including a variety of cancers [7].

## 2. Lipid Metabolism

According to the International Lipid Classification and Nomenclature Committee, lipids are currently classified into eight categories: (1) fatty acids; (2) glycerolipids; (3) glycerophospholipids; (4) sphingolipids; (5) sterol lipids; (6) prenol lipids; (7) saccharolipids; and (8) polyketides [8]. In the cells, the structure of lipids determines their function and metabolic fate [1,2]. Lipids that are currently understood as most relevant to cancer development and chemotherapy are fatty acids, glycerolipids, glycerophospholipids, sphingolipids, and sterol lipids.

Fatty acids composed of a hydrocarbon chain with one terminal carboxyl group (COOH) are produced by fatty acid synthases from acetyl-CoA and malonyl-CoA precursors, by lipases in the degradation of glycerolipids or by phospholipase A_1_, A_2_ and B in the breakdown of glycerophospholipids. The degradation of fatty acids via β-oxidation leads to the release of energy (large quantities of ATP) and generates reactive oxygen species [9,10]. Glycerolipids, fatty acid esters of glycerol (mono-, di-, or tri-glyceride), are biosynthesized by the *sn*-glycerol-3-phosphate pathway which predominates in liver and adipose tissue and the monoacylglycerol pathway in the intestines [11].

**Figure 1:**
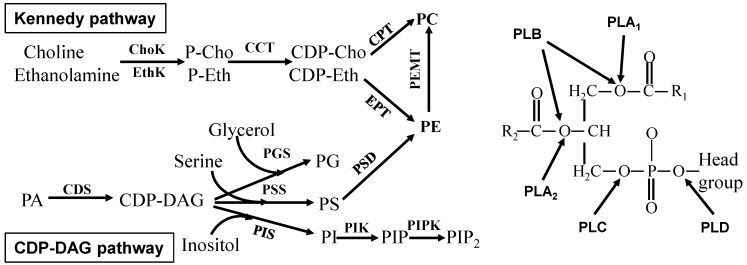
Glycerophospholipid metabolism. Left, glycerophospholipid synthesis; Right, glycerophospholipid degradation. The enzymes are choline kinase (ChoK), ethanolamine kinase (EthK), cytidine 5'-triphosphate (CTP)-phosphocholine (or phosphethanolamine) cytidylyltransferase (CCT), cholinephosphotransferase (CPT), ethanolaminephosphotransferase (EPT), phosphatidylethanolamine *N*-methyltransferase (PEMT), CDP-diacylglycerol synthase (CDS), phosphatidylglycerol synthase (PGS), phosphatidylserine synthase (PSS), phosphatidylserine decarboxylase (PSD), phosphatidylinositol synthase (PIS), phosphatidylinositol kinase (PIK), phosphatidylinositol phosphate kinase (PIPK), phospholipase A_1_ (PLA_1_), phospholipase A_2_ (PLA_2_), phospholipase B (PLB), phospholipase C (PLC), and phospholipase D (PLD). P-Cho, phosphocholine; P-Eth, phosphoethanolamine; CDP-Cho, CDP-choline; CDP-Eth, CDP-ethanolamine; PA, phosphatidic acid; PC, phosphatidylcholine; PE, phosphatidylethanolamine; PG, phosphatidylglycerol; PS, phosphatidylserine; PI, phosphatidylinositol; PIP, phosphatidylinositol phosphate; PIP2, phosphatidylinositol bisphosphate; R_1_ and R_2_, acyl group; and Head groups are choline, inositol, serine, ethanolamine or glycerol.

Glycerophospholipids are the main component of biological membranes and contain at least one *O*-1-acyl, *O*-1-alkyl, or *O*-1-alkenyl residue attached to the glycerol moiety. The presence of an additional head group (such as choline, ethanolamine, serine, inositol, and glycerol) attached to the phosphate allows for many different glycerophospholipids. Both biosynthesis (CDP-DAG pathway and Kennedy pathway) and degradation (different phospholipases) of glycerophospholipids are regulated by different signaling pathways (Figure 1). Interestingly, many different bioactive lipid molecules, such as inositol trisphosphate, diacylglycerol, arachidonic acid, phosphatidic acid, and lysophosphatidic acid, are generated during glycerophospholipid metabolism, and these bioactive lipid molecules in turn regulate different signaling pathways in the cells [12,13]. The metabolism of glycerophospholipids is very complex and it is not fully understood how and when their substitutions and modifications occur.

**Figure 2:**
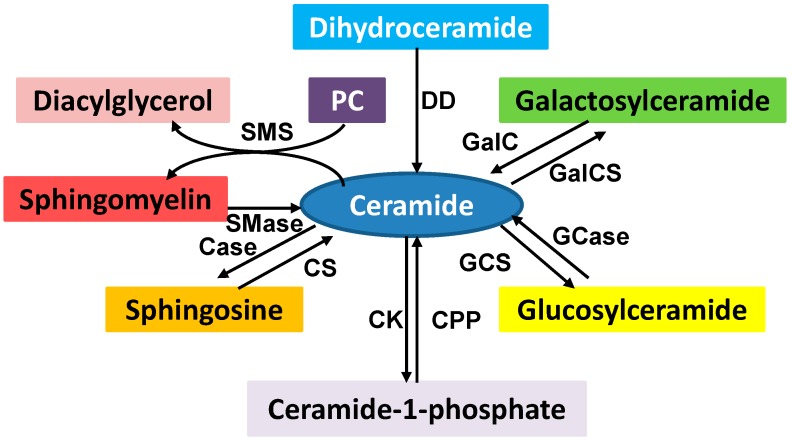
Sphingolipid metabolism. Ceramide is a key intermediator in sphingolipid metabolism. The enzymes involved in sphingolipid metabolism are ceramidase (Case), ceramide kinase (CK), ceramide-1-phophosphate phosphatase (CPP), ceramide synthase (CS), dihydroceramide desaturase (DD), galactosylceramide synthase (GalCS), galactocer (GalC), glucosylceramide synthase (GCS), glucosylceramidase (Gcase), sphingomyelinase (Smase), and sphingomyelin synthase (SMS). Many of these products play an important role in cell signaling which regulates a variety of cellular functions. SMS converts phosphatidylcholine (PC) and ceramide to sphingomyelin and diacylglycerol which brings two major classes of lipids in cell metabolism and signaling.

Sphingolipids, including the sphingomyelins and glycosphingolipids, are *de novo* synthesized in the endoplasmic reticulum (ER) from nonsphingolipid precursors [14]. Sphingomyelins can be hydrolyzed by sphingomyelinases to produce ceramides and phosphocholine. The conversion of sphingosine to sphingosine-1-phosphate, sphingosine to ceramide, ceramide to ceramide-1-phosphate, and ceramide to glucosylceramide is catalyzed by different enzymes (Figure 2). Sphingolipids are also structural components of cell membrane, and the products of sphingolipid metabolism such as ceramide, ceramide-1-phosphate, sphingosine, sphingosine-1-phosphate, and glucosylceramide act as bioactive lipid molecules in apoptotic and drug-resistant signaling.

Sterol lipids, such as cholesterol, are biosynthesized in a highly complex series of at least thirty different enzymatic reactions to form four linked hydrocarbon rings (hexagons and pentagon) and are not readily biodegradable. Cholesterol is an integral component of cellular membranes, determines membrane rigidity and fluidity, and plays a crucial role in membrane organization, dynamics, and function [15]. Some steroid lipids, such as vitamins, testosterone, estrogen, and cortisone are ligands and can regulate cell signaling to control a myriad of bodily functions. Recently, more and more data supports that the levels of cellular cholesterol are significantly increased in cancer cells and tissues, and cholesterol promotes cell proliferation, tumor progression, and drug resistance [16,17].

Prenol lipids, Saccharolipids and Polyketides are produced mainly in bacteria, fungi and plants [18,19,20]. Some of these lipids and their derivatives are vitamins or antimicrobial, antiparasitic, and anticancer agents. For instance, vitamins A, E, and K belong to prenol lipids, lipopolysaccharides are one of the most familiar saccharolipids [19], and erythromycins, tetracyclines, avermectins, and epothilones are polyketides or their derivatives [21].

Lipoproteins are not classified as lipids but are a group of biochemical assemblies that contains both proteins and lipids, covalently or non-covalently bound to the proteins, which allow fats to move through the water inside and outside of the cells. The proteins serve to emulsify lipid molecules. Most importantly, lipoproteins can be enzymes, transporters, structural proteins, antigens, adhesins, or toxins that can regulate cellular functions including apoptosis [22].

## 3. Regulation of Lipid Metabolism

**Figure 3:**
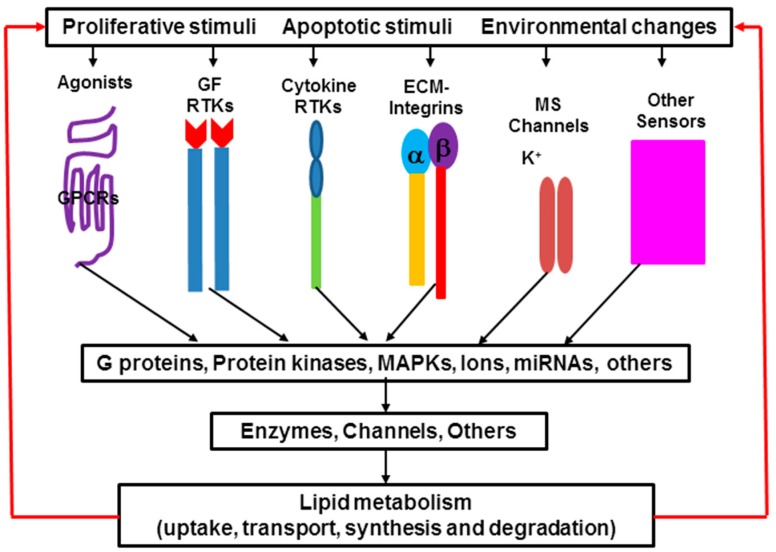
Signaling transduction in lipid metabolism. Extracellular signals induce different pathways that regulate lipid metabolism in the cells. Protein kinases include protein kinase A, B and C; mitogen-activated protein kinases (MAPK) includes extracellular signal-regulated kinases (ERK), p38 kinase and c-Jun *N*-terminal kinases (JNK); Enzymes that are involved in lipid uptake, transport, synthesis and degradation; Ions: Ca^2+^, K^+^, Na^+^, H^+^. Some bioactive lipid molecules are ligands, and in turn induce different signaling pathways. MS, mechanical stress.

Lipid metabolism, including lipid uptake, transport, synthesis, and degradation, is a complex process. Biosynthesis and degradation of different lipids are regulated by different signaling pathways, and the same lipid can be regulated by different signaling pathways in different tissues and cells as well as under physiological, pathophysiological or therapeutic conditions [1,2]. Either activation or inhibition of these signaling pathways is based on cell needs and responds to environmental changes (Figure 3). There are more than one hundred enzymes regulating lipid metabolism in the cells. Recent studies show that expression of many of these enzymes is regulated by microRNA (miRNA). It indicates that miRNA also plays an important role in lipid metabolism. Alteration of lipid metabolism leads to the changes of membrane compositions, protein distribution and function, gene expression, and cellular functions, and further causes the development and progression of many diseases such as inflammation, hypertension, diabetes, liver disease, heart disease, renal disease, neurological disorder, cystic fibrosis, and cancer [3,23]. On the other hand, manipulation of lipid metabolism can lead cancer cells to apoptosis.

### 3.1. Signaling Pathways in Lipid Metabolism

**G protein-coupled receptors (GPCRs):** GPCRs are a superfamily of receptors that are vital in a wide array of physiological processes and are the most important class of membrane proteins in clinical medicine accounting for approximately 40% of all current therapeutics [24,25]. Allosteric ligands bind to GPCRs leading to the activation of G protein and downstream enzymes involved in lipid uptake, transport, synthesis, and degradation. Over the past three decades, research has been not only centered on the identification of GPCR-signaling that regulates lipid metabolism but has also demonstrated that some bioactive lipid molecules, such as lysophosphatidic acid, sphingosine-1 phosphate, free fatty acids, and platelet activating factors, are ligands for activating GPCR-signaling [26,27,28].

**Tyrosine kinases:** Cytokines and growth factors exert their biological effects by binding to specific cell surface receptors on target cells. Most of these receptors have a tyrosine kinase activity domain that is localized at the cytoplasmic region of the molecule [29,30]. The interaction of the cytokines and growth factors with the receptors induces the kinase activity of the receptor, and further activates downstream effectors such as protein phosphorylation and enzyme activation. Cytokine signaling pathways respond to innate immunity and inflammation which can induce phospholipases (A_2_, C, and D), sphingomyelinases, and the enzymes that regulate cholesterol metabolism [31,32]. Mitogenic signaling carried out by growth factors regulates cell growth and proliferation which is involved in the activation of many lipid-metabolism-related enzymes [29,32]. Recently, a large body of evidence has indicated that agonists of some GPCRs can activate growth factor receptor tyrosine kinases in the absence of growth factor [33,34]. The transactivation by GPCRs also links to cellular lipid synthesis and degradation.

**Integrin signaling:** Integrin signaling governs cellular adhesion and transmits signals leading to the activation of intracellular signaling pathways aimed to prevent apoptosis. This regulation is associated with lipid metabolism. Integrin-associated Lyn kinase can promote cell survival by suppressing acid sphingomyelinase activity [35]. In bovine pulmonary artery endothelial cells integrin signaling causes arachidonic acid release by membrane translocation and phosphorylation of cytosolic phospholipase A_2_ as well as tyrosine phosphorylation of the mitogen-activated protein kinase (MAPK) [36]. Tyrosine kinase Btk regulates E-selectin-mediated integrin activation and neutrophil recruitment by controlling phospholipase C γ2 and phosphatidylinositol-3 Kinase γ pathways [37]. Integrin signaling also links large integrin-associated intracellular protein complexes, which act as anchors for the cytoskeleton and as signaling hotspots, and regulates trafficking of cholesterol-enriched membrane microdomains known as lipid rafts [38,39].

**Ion-channel signaling:** Cells commonly use membrane lipids to modulate the function of ion channels. The interaction of ion channels with membrane lipids can be highly specific and is often important for full functional and structural integrity of ion channels [40]. Recent studies indicate mechanistic insights into how lipid modification (for example palmitoylation) controls large conductance calcium- and voltage-activated potassium channel trafficking and cross-talk with phosphorylation-dependent signaling pathways [41]. In addition, a constantly growing body of literature reveals that some agonists, despite their direct effect on ion channels, may also influence functions of ion channels via cellular lipid metabolism. For instance, the conversion of sphingomyelin to ceramide or phosphatidylinositol bisphosphate to diacylglycerol not only contributes to the membrane surface potential, but also affects the functional properties of some channels (from the opened state to the closed state of the channels) [42,43].

**Other signaling:** Signaling pathways that respond to environmental changes such as pH changes, oxidative stress, and mechanical stress also play an important role in lipid metabolism. The pH-dependent group II phospholipase A_2_ enhances membrane phospholipid degradation which contributes to chemical hypoxic and ischemic injury of rat hepatocytes [44], and pH also activates phospholipase C during intracellular infection [45]. Oxidative stress induces arachidonate release from human lung cells [46] and redox-active antioxidant modulation of lipid signaling in vascular endothelial cells by the regulation of phospholipase D, phospholipase A_2_, lipoxygenase, and cyclooxygenase [47]. Mechanical forces generated from blood pressure and blood flow are responsible for lipid metabolism through the activation of phospholipases A_2_, C, and D, phosphatidylinositol 3-kinase, sphingomyelinase, and phosphatidylcholine biosynthesis via a variety of signaling pathways [48]. Sterols, bile acids, and fatty acids are the endogenous ligands of many nuclear receptors such as orphan receptor, farnesoid X receptor, peroxisome proliferator-activated receptor, vitamin D receptor, constitutive androstane receptor, and pregnane X receptor [49]. These receptors coordinately regulate lipid, glucose, energy, and drug metabolism [50]. Many non-enzymatic proteins, such as p53, caveolin, and cell-death-inducing DNA fragmentation factor 45-like effector, are also involved in lipid metabolism [51,52]. The signaling network of lipid metabolism can activate different kinases and a variety of other enzymes that regulate different cell functions.

### 3.2. Enzymes in Lipid Metabolism

Fatty acid synthase, choline kinase, ceramide synthase, phosphatidylinositol-3 or -4 kinases, and the enzymes that catalyze the generation of fatty acids, phospholipids, and cholesterol all play integral roles in lipid biosynthesis which can alter lipid compositions in cellular membranes and cell fates by regulating cellular functions. Fatty acid synthase and choline kinase are overexpressed in many cancer cell lines and tumors [53,54]. Understanding the regulation of these enzymes in cancer cells not only to explore how they contribute to cancer cell growth, proliferation and tumor progression, but also to evaluate whether their specific inhibitors can be used for cancer chemotherapy [55]. The levels of cellular cholesterol are much higher in cancer cell lines and tissues than their normal compartments [56,57,58,59]. The data indicate that overexpression of enzymes in cholesterol synthesis or activation of these enzymes occurs in cancer cells. Cholesterol is capable of promoting cell proliferation, migration, tumor progression, and chemotherapy resistance [16,17]. Cholesterol-lowering agents could induce cancer cell apoptosis and exhibit important antitumor activity [60,61,62,63,64,65].

Phospholipases A_2_, C, and D, sphingomyelinases and ceramidase are key enzymes in the regulation of lipid degradation during cell response to stimuli. Phospholipase A_2_-mediated phospholipid degradation generates free fatty acids and accompanies with the generation of lysophospholipids which are emerging as a novel class of inflammatory lipids [1,2]. Phospholipase C hydrolyzes glycerophospholipids to generate diacylglycerol which uniquely functions as a basic component of membranes, an intermediate in lipid metabolism, and a key element in lipid-mediated signaling [66]. Inositol trisphosphate, one of the products of phospholipase C in the hydrolysis of polyphosphoinositides, binds to inositol-trisphosphate receptor and evokes intracellular Ca^2+^ signaling for normal cell survival and is also actively involved in apoptosis induction and progression [67]. Phospholipase D catalyzes the hydrolysis of the terminal diester bond of glycerophospholipids with the formation of phosphatidic acid plus head groups. Phosphatidic acid regulates the activity of small GTPases or directly binds some small GTPases to membranes [68]. Recent studies show that phosphatidic acid directly interacts with mammalian target of rapamycin (mTOR) in a manner that is competitive with rapamycin and is required for the stability and kinase activity of both mTOR complexes (mTORC1 and mTORC2) [69,70]. Phospholipase d-phosphatidic acid-mTOR signaling leads to podocyte hypertrophy and apoptosis [71]. Sphingomyelinases hydrolyze sphingomyelin to produce ceramide which can also be synthesized *de novo* by ceramide synthase. Ceramide was the first lipid molecule linked to cell death signaling about two decades ago and has received considerable attention as a key regulator of programmed cell death [72,73].

Many enzymes, such as cyclooxygenase, lipoxygenase, prostaglandins synthase, diacylglycerol kinase, phosphatidate phosphatase, and glucosylceramide synthase, are also very important in the cells because they can convert one lipid molecule to another in the cells (Figure 1 and Figure 2) [1,2,3,4,5]. For instance, ceramide can be converted to sphingosine, ceramide-1-phosphate, and glucosylceramide by ceramidase, ceramide kinase, or glucosylceramide synthase [74,75]. These converted derivatives are also important signaling molecules but play different roles in the regulation of cellular functions [72,73,74,75].

### 3.3. MicroRNAs (MiRNAs) in Lipid Metabolism

MiRNAs are small, evolutionarily conserved, and non-coding RNA molecules (containing about 22 nucleotides) that can regulate gene expression at the posttranscriptional level. To date, more than 30 miRNAs, including miRNA-122, miRNA-33, and miRNA-370, have been discovered to play important roles in the regulation of lipid metabolism such as fatty acid oxidation, cholesterol efflux, and the biosynthesis of fatty acids, cholesterol, and triacylglycerol [76,77,78,79].

MiRNA-122 was initially identified as a highly abundant miRNA (~70% of total miRNA) in the liver [80]. This conserved, liver-specific miRNA has been associated with the regulation of liver metabolism, including the biosynthesis of fatty acids, triacylglycerol, and cholesterol, which can stimulate the production of endoplasmic reticulum-associated lipid droplets and cholesterol-rich membrane domains (lipid rafts or caveolae) [81,82]. MiRNA-122 also promotes the propagation of hepatitis C virus by multiple mechanisms, has been shown to be down-regulated in hepatocellular carcinoma, and plays a role in fatty liver disease [81,83]. MiRNA-33 is highly conserved across species and can be found in numerous cell types, including macrophages, hepatocytes, and endothelial cells. It has two isoforms (**a** and **b**) and is an intronic miRNA located in a non-coding region of sterol-regulatory binding factor (*SRBF*) genes which are involved in cholesterol uptake and synthesis [84]. MiRNA-33, which targets ATP-binding cassette transporter subfamily A1 (*ABCA*) gene controlling the movement of cholesterol out of the cell, is also a key posttranscriptional regulator of cellular cholesterol homeostasis [85]. MiRNA-33a and miRNA-33b also contribute to the regulation of fatty acid metabolism (β-oxidation) by modulating the expression of carnitine palmitoyltransferase1A, carnitine *O*-octanyltransferase, and hydroxylacyl-CoA dehydrogenase-3-ketoacyl-CoA thiolase [86]. MiRNA-33 is responsive to alterations in cholesterol levels associated with diet or medication, making miRNA potential biomarkers of response to environmental stimuli and targets of therapeutic interventions. Recent studies showed that miRNA-370 has similar effects on lipid metabolism as miRNA-122. MiRNA-370 controls the expression of miRNA-122, targets and regulates lipid metabolism by up-regulating multiple genes coding for *SRBF* 1c, diacylglycerol O-acyltransferase 2, fatty acid synthase, and acyl-CoA carboxylase 1 [87]. MiRNA-370 also targets carnitine palmitoyl transferase which mediates the transport of long-chain fatty acids across the membrane and fatty acid oxidation [76]. MiRNA-378, another intronic miRNA located within the genomic sequence of peroxisome proliferator-activated receptor γ coactivator-1α (a master regulator of energy metabolism), also plays important roles in regulating lipid metabolism by targeting estrogen-related receptors and GA-binding protein α in adipocyte differentiation and lipid synthesis [88]. MiRNA-106, -144 and -758 regulate *ABCA1* expression involved in cholesterol metabolism. MiRNA-96, -125, -185, -223 and -455 have been recently described to regulate *SRBF 1* expression and HDL (high-density lipoprotein) uptake [76,77]. MiRNA-1, -143, -206 and -371 can promote adipogenesis [78]. In contrast, miRNA-27, -130, -206, and -369 negatively regulate adipocyte differentiation [76,77,78,79].

## 4. Lipid and Apoptotic Signaling

Cell death that maintains organismal and cellular homeostasis has been defined as an irreversible loss of plasma membrane integrity which is associated with changes in membrane lipid metabolism. Three types of cell death can be distinguished in mammalian cells according to morphological criteria: autophagy, necrosis and apoptosis [89]. Cell death, either progressive or acute, is also a hallmark characteristic of cancer treatment and various diseases, including cardiac disease, brain injury, and renal failure [90].

### 4.1. Cell Death and Regulation

Autophagy is an evolutionarily conserved catabolic pathway that allows cells to degrade and recycle cellular components. This process mainly maintains a balance between the manufacture of cellular components and the breakdown of damaged or unnecessary organelles and cellular constituents. Disruption of autophagy is involved in diverse human diseases including cancer. Autophagy is death receptor-independent, and target of rapamycin (TOR) acts as an efficient gatekeeper, on which it exerts an inhibitory effect [91]. Necrosis is an accidental and uncontrolled form of cell death lacking underlying signaling events. The factors that cause necrosis are external to the cells or tissues, such as physical damage (mechanical stress and detergent-induced cytolysis), infection, toxins, or trauma, which can lead to cell injury and result in the unregulated digestion of cell components and the premature death of cells. Necrosis is often associated with pathological conditions such as injury of organs. There are two main necrotic pathways: the death receptor pathway which is stimulated by tumor necrosis factor (TNF) α, Fas ligand, and TNF-related apoptosis-inducing ligand (TRAIL) and the mitochondrial pathway that leads to the generation of reactive oxygen species, ATP depletion, the accumulation of H^+^ and acidosis, and mitochondrial dysfunction [92]. A relatively new form of necrosis, termed necroptosis or programmed necrosis, has been identified. It exhibits the features of necrosis and apoptosis, and is caspase independent but receptor interaction protein kinase dependent [93].

Apoptosis, or so-called programmed cell death, is a type of cell death that is not involved in an inflammatory response and occurs in a tightly controlled manner. In contrast to autophagy and necrosis, apoptotic signaling is triggered either by death receptor (extrinsic pathway) or by mitochondria (intrinsic pathway) [94,95]. The extrinsic pathway is that death receptor activated by TNF-α, Fas ligand (CD95/APO1) or TRAIL leads to the assembly of a death-inducing signaling complex formed by the death receptors, adapter proteins, and caspases (cysteinyl aspartate specific proteases) such as caspase-8 and caspase-10. Caspase-8 directly triggers caspase-3 activation or can interact with the intrinsic apoptotic pathway by cleaving Bid (a pro-apoptotic member of the Bcl-2 (B-cell lymphoma 2) family) to form the truncated Bid (tBid) which translocates to the mitochondria and results in the release of cytochrome c. The intrinsic pathway can be induced by a variety of upstream receptor-independent stimuli, such as anticancer drugs, toxins, and radiation, and causes the alteration of mitochondrial membrane property and function. The permeabilization of mitochondrial outer membrane results in the release of cytochrome c. Cytochrome c interacts with apoptotic protease activating factor 1 (Apaf-1) and pro-caspase-9 to form a caspase activation complex, the apoptosome. The response to death receptor and the permeabilization of mitochondrial membrane are directly associated with lipid metabolism.

### 4.2. Bioactive Lipid Molecules and Apoptosis

As described above, many bioactive lipid molecules play an important role in the regulation of many different cell functions. Here we discuss the bioactive lipid molecules that are produced in lipid metabolism and are associated with apoptosis.

#### 4.2.1. Fatty Acids

Fatty acids can induce apoptosis in different cell types [96,97,98,99]. Short chain fatty acids (C2–5) inhibit histone deacetylases, resulting in a hyperacetylation of core histone proteins [53]. Hyperacetylation of histones is associated with transcriptional regulation and growth inhibition in colonic epithelial cells [54]. Long-chain fatty acids induce ER stress which in turn activates c-jun *N*-terminal kinase (JNK) and CEBP (CCAAT/enhancer binding protein)-homologous protein (CHOP). JNK leads to the up-regulation of the pro-apoptotic BH3 (Bcl-2 homology domain 3) only proteins p53 (tumor protein p53)-upregulated modulator of apoptosis (PUMA). CHOP enhances the expression of the proapoptotic BH3-only protein Bim, contributes to PUMA up-regulation, and mediates the generation of reactive oxygen species (ROS). Bim, in cooperation with PUMA, induces the activation of the multi-domain executioner proapoptotic protein Bax. Bax activation results in mitochondrial membrane permeabilization, activation of the caspase cascade and leads to cell death [100].

Oxidation of fatty acids is the source of increased production of mitochondrial ROS [10]. At low levels ROS is a signaling molecule, while at high levels it can damage organelles, particularly the mitochondria. The toxicity of fatty acid oxidation is related to both the chain length and the degree of unsaturation. The longer the chain is and the more unsaturated the species, the more toxic it is [101]. 4-Hydroxy-2-nonenal (HNE), a major α,β-unsaturated aldehyde product of n-6 fatty acid oxidation, is a highly toxic and most abundant stable end product of lipid peroxidation, and has been considered as an oxidative stress marker [102]. ROS can cause unwanted stress that can activate stress-activated protein kinase or JNK [102]. Oxidative damage and the associated mitochondrial dysfunction result in energy depletion, accumulation of cytotoxic mediators, modulation ligand-independent signaling by Fas (CD95) receptor, and caspase activation which lead to apoptosis. Fatty acids also activate AMP (adenosine monophosphate)-activated protein kinase [103], extracellular signal-regulated kinase (ERK) [104], GPCR signaling [105], Toll-like receptor 4/NF-κB [106], Src-JNK [107], and protein kinase C [108] signaling as well as sphingomyelinase-ceramide signaling [109] in the regulation of apoptosis. These data clearly demonstrate that oxidation of fatty acids has been implicated in the tissue damage, dysfunction, and injury associated with aging and other pathological states such as cancer, metabolic diseases, neurodegenerative diseases, cardiovascular and inflammatory complications [102,110].

#### 4.2.2. Phosphatidic Acid

Phosphatidic acid is a key intermediate in glycerophospholipid metabolism. Earlier studies showed that phosphatidic acid is a signaling molecule with growth factor-like properties, inducing DNA synthesis and cell proliferation [111] and stimulating phospholipase C activation and calcium release [112] in the cultured cells. Later, phosphatidic acid, as a second messenger, can activate NADPH (nicotinamide adenine dinucleotide phosphate) oxidase in human polymorph nuclear leukocytes [113], which could link to apoptotic signaling. Recent evidence supports the involvement of phosphatidic acid in apoptotic signaling. For instance, cerium activates phospholipase D and produces phosphatidic acid which induces the biphasic burst of superoxide anions and regulates MAPK (mitogen-activated protein kinases)-mediated apoptosis [114]. Increasing levels of production of phosphatidic acid on the mitochondrial surface results in mitochondrial aggregation and facilitates the fusion process [115]. Phosphatidic acid, one of the major acidic phospholipids found in lysosome membrane, is essential for tBid-induced lysosomal membrane permeabilization and links to the lysosomal-mitochondrial mediated apoptotic pathway [69]. Galectin-8, a potent pro-apoptotic agent, induces phospholipase D/phosphatidic acid signaling pathway that enhances ERK-mediated apoptosis in Jurkat T cells [116]. We recently found that phosphatidic acid, produced by shear stress-induced phospholipase D activation, stimulates mTOR signaling, and causes podocyte hypertrophy and apoptosis [71]. Taken together, phosphatidic acid plays an important role in the regulation of apoptotic signaling.

#### 4.2.3. Ceramide

Ceramide is capable of triggering apoptosis in almost any cell, including tumor cells. Ceramide can be generated by a *de novo* pathway (ceramide synthase) or by sphingomyelinases in response to various stress stimuli, such as cytokines, heat shock, growth factors, vitamin D, TNF-α, CD95/Fas, chemotherapeutic agents, toxin, irradiation, UV-light, and infection by different signaling pathways [72]. Elevation of cellular ceramide levels directly or indirectly regulates the activities of a number of enzymes and signaling components, including MAP kinases, ceramide-activated kinase, ceramide-activating serine/threonine phosphatases such as protein phosphatase 1A and 2A, protein kinase C ζ, phospholipases such as phospholipase A_2_ or D, CPP32-like caspases, cathepsin D, transcription factors such as NF-κB, and kinase suppressor *ras* [117,118,119,120,121,122]. These enzymes and signaling components play an important role in the regulation of apoptotic signaling. On the other hand, an irreversible step in apoptotic processing is mitochondrial outer membrane permeabilization which releases critical proteins such as cytochrome c. The channels for protein release are controlled by Bcl-2 family proteins based on cell physiological function: anti-apoptotic proteins (Bcl-x, Bcl-w, and others) destabilize the channels whereas pro-apoptotic proteins (Bax, BAD, Bak, Bok, and others) act synergistically with ceramide to increase membrane permeability [123]. Ceramide can self-assemble in the mitochondrial outer membrane to form large stable channels capable of releasing cytochrome c [124]. Cytochrome c further interacts with Apaf-1, activates several caspases and forces cell to undergo apoptosis. The role of ceramide in apoptosis indicates that ceramide could be a potential anticancer drug.

#### 4.2.4. Cholesterol

Cholesterol modulates cell signaling through the cholesterol–protein interaction, cholesterol–phospholipid interaction, and membrane dynamics. Increasing cholesterol levels promotes cell proliferation, tumor progression, and chemotherapy resistance [16,17]. However, recent studies show that cholesterol is also involved in apoptotic signaling. A significant fraction of cholesterol that accumulates in atherosclerotic lesions is oxidized to yield a number of derivatives, called oxysterols. Cholesterol oxidation produces 7α-hydroxy-, 7β-hydroxy-, 7-keto-, 20-hydroxy-, and 25-hydroxycholesterol, and can also attack the Δ5 double bond of cholesterol, forming cholesterol-5,6-epoxide which could react spontaneously with nucleophiles and behave like alkylating agents with direct carcinogenic properties [125]. Oxysterols increase intracellular levels of ROS, induce modification of cellular proteins (pro- and anti-apoptotic molecules), and alter gene expression and mitochondrial membrane properties. It is clear that accumulation of oxysterols may strongly stimulate the mitochondrial pathway of apoptosis [126]. On the other hand, toxic amyloid beta peptides (Aβ) are overproduced and accumulate in mitochondrial matrix of experimental models of Alzheimer’s disease. Specific mitochondrial cholesterol pool sensitizes to Aβ-induced oxidant cell death and caspase-independent apoptosis by cholesterol-mediated perturbation of mitochondrial membrane dynamics [127]. Recent investigations have shown that dendrogenin A is a selective inhibitor of cholesterol epoxide hydrolase and it triggered tumor re-differentiation and growth control in mice and improved animal survival [128]; cholesterol-5,6-epoxide metabolites, moreover, contribute to the anticancer pharmacology of Tamoxifen [129]. Although cholesterol promotes cell proliferation, oxidized cholesterol can lead cells to apoptosis.

#### 4.2.5. Apolipoproteins

Some apolipoprotein Ls (ApoL1 and ApoL6) share structural and functional similarities with Bcl-2 family proteins that play crucial roles in regulating apoptosis. ApoL1 is inducible by p53 in p53-induced cell death [130], and overexpression of ApoL6 induces the release of cytochrome c and Smac/DIABLO from mitochondria and activation of caspase-9 via a mitochondria-mediated pathway [131]. The levels of apolipoprotein E (ApoE) mRNA and protein are up-regulated during staurosporine-induced apoptosis and are also correlated with increased caspase-3 activity and apoptotic morphological changes [132]. ApoE synthesis induced by neuronal damage or stress indicates its neurotoxic effect and is associated with apoptotic signaling [133]. The genesis of atherosclerosis is also associated with lipoprotein oxidation [126]. The oxidized low-density lipoprotein could enhance arterial apoptosis via mitochondrial and death receptor pathways [134]. The oxidative stress has also been implicated in the cardiovascular complications in chronic renal failure patients [133].

#### 4.2.6. Intracellular Calcium

Ca^2+^ is not a lipid but has strong correlations with lipid metabolism and cell death. Intracellular calcium homeostasis is crucial for healthy cells, and the disruption of intracellular calcium homeostasis will cause cell damage, and even death [135,136]. In mammalian cells, the endoplasmic reticulum (ER) forms the main intracellular Ca^2+^ reservoir. Intracellular Ca^2+^ can be mobilized from ER by inositol trisphosphate (IP_3_). IP_3_ is produced by phospholipase *C*-hydrolyzed phosphatidylinositol bisphosphate and can bind to IP_3_ receptor on the ER [137]. Extracellular Ca^2+^ enters to the cells controlled by membrane transporter such as plasma membrane calcium ATPases and channels such as Trp. These transporters and channels are regulated by lipids [126]. Apoptotic cells rely on increased intracellular Ca^2+^ concentrations [135], mediated by the release from ER and by capacitive Ca^2+^ influx through transporters or channels [137,138,139]. Mitochondrial uptake of Ca^2+^ causes ATP production, mitochondria outer membrane permeabilization and release of cytochrome c [135]. Recently, intracellular organelles coordinate complex molecular mechanisms in the regulation of Ca^2+^ signaling and lipid metabolism. A number of experimental evidence support that cell apoptosis regulated by alteration of intracellular Ca^2+^ homeostasis is associated with the tight interplay between ER and mitochondria known as the mitochondria-associated membrane (MAM) [140,141,142,143]. In mammalian cells, the formation of these contact sites appear to be required for key cellular events including rapid transmission of calcium from the ER to mitochondria, the import of phosphatidylserine into mitochondria from the ER for decarboxylation to phosphatidylethanolamine, the formation of autophagosomes, and the regulation of the morphology, dynamics and functions of mitochondria, and cell survival [144]. In a mouse model of the human lysosomal storage disease, GM1-ganglioside is accumulated in the glycosphingolipid-enriched microdomain (GEM) fractions of MAMs [145]. Meanwhile, the MAM fractions from rat liver contain highly active sphingolipid-specific glycosyltransferases [146].

## 5. Lipid Metabolism, Cancer Treatment and Drug Resistance

Cancer is characterized by uncontrolled cell growth with increased proliferation and decreased apoptosis and enhances migrating behavior of cells by promoting their ability to invade adjacent tissues and/or metastasize to non-adjacent organs and tissues. Cell proliferation requires duplication of all macromolecular components during each cell division. Aberrant lipid metabolism is now recognized as one of the key features of cancer cells because cell proliferation requires increased lipid biosynthesis, and lipid catabolism produces bioactive molecules which act as signal molecules to regulate cancer metastasis [1,2,7].

### 5.1. Lipid Metabolism in Cancer

Three classical lipids, fatty acids, phospholipids and cholesterol, are dramatically increased and actively biosynthesized in cancer cells and tumors. At first, evidence shows that expression and activity of fatty acid synthase are extremely low in nearly all nonmalignant adult tissues, whereas it is significantly up-regulated in a number of solid and aggressive cancers [147]. Fatty acids are also building blocks for glycerolipids, glycerophospholipids, and other lipids. Secondly, expression of choline kinase, a key enzyme in biosynthesis of phosphatidylcholine, is up-regulated in a variety of cancer cell lines and tumors, and choline kinase can be activated by different growth factors and oncogene-coding proteins such as *ras* [148]. Thirdly, active sterol biosynthesis remains an essential metabolic component of cell proliferation. Up-regulating cholesterol biosynthesis and cholesterol efflux are only discovered in proliferating normal tissues and tumors [15,16,17]. Transcriptional profiling by microarray has demonstrated that refractory cancers exhibit significant overexpression of a number of genes in cholesterol biosynthetic pathway [56]. Cholesterol biosynthesis happens much earlier than DNA synthesis, and inhibiting cholesterol biosynthesis slows cell growth, suggesting a linkage between the cholesterol and DNA synthetic pathways [149]. Lipid metabolism in cancer cells remains largely unknown. Recently, lipidomics (also called lipid profiling) has provided more details of lipid metabolism by comparing lipid profiles of normal and cancer cells or tissues [150,151], which could be useful for identifying clinical biomarkers for earlier diagnosis, and allow evaluation of determining the efficacy of cancer therapy.

### 5.2. Anticancer Drugs, Lipid Metabolism and Apoptosis

A number of anti-cancer drugs are lipid-based or effective in terms of their ability to regulate lipid metabolism. Many anticancer drugs, such as cytarabine, daunorubicin, doxorubicin, etoposide, fludarabine, irinotecan, paclitaxel, tamoxifen, taxol, vinblastine, and vincristine, can impact ceramide accumulation by inducing ceramide synthase to catalyze *de novo* ceramide synthesis or by activating sphingmyelinase to catalyze sphingomyelin degradation [117,152]. Based on the structure of ceramide, ceramide analogs such as ceramidoids, 4, 6-diene-ceramide, and C_16_-serinol are also used as anticancer drugs [117]. Some anticancer drugs target fatty acid synthesis by inhibiting fatty acid synthase [153], phospholipase A_2_ [154], and lipases [155]. Some anticancer drugs can significantly reduce the levels of cellular cholesterol by blocking different steps of cholesterol biosynthesis [16,17,156]. Some anticancer drugs are developed based on blocking the conversion of lipid products [75]. In each case, anticancer drugs cause the alternations of lipid metabolism in cancer cells and the result leads to cancer cells to growth arrest and/or apoptosis.

**Figure 4:**
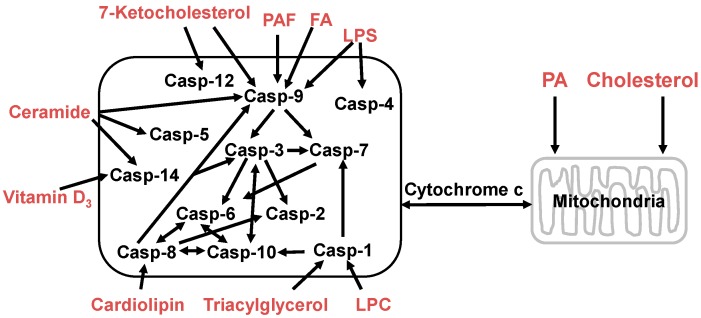
Lipids regulate apoptotic signaling in the cells. Phosphatidic acid (PA) and cholesterol can modulate mitochondrial membrane permeability and triggers apoptosis via the lysosomal-mitochondrial pathway; and the bioactive lipid molecules, such as platelet-activating factor (PAF), fatty acids (FA), lysophosphatidylcholine (LPC), lipopolysaccharide (LPS), 7-ketocholesterol, ceramide, vitamin D3, cardiolipin, and triacylglycerol, can induce apoptotic signaling pathways by activating different caspases. The released cytochrome c in mitochondrial pathway interacts with Apaf-1 and pro-caspase-9 to form a caspase (Casp) activation complex, the apoptosome, or caspase activation can cleave Bid (a pro-apoptotic member of the Bcl-2 family) to form the truncated Bid (tBid) which translocates to the mitochondria and results in the release of cytochrome c.

Some lipids directly induce caspase activation leading to programmed cell death. For example, triglyceride [157], lysophosphatidylcholine [158], lipopolysaccharide [159], and cholesterol [160] can induce or trigger caspase-1 activation. Apoptosis induced by fatty acids and their derivatives is associated with significant activation of caspase-2, -3, -6, -7, -8 and -9 [161,162]. Cardiolipin is a mitochondria-specific phospholipid and provides an essential activating platform for caspase-8 on mitochondria [163]. The 7-Ketocholesterol activates caspases-3, -7, -8, and -12 in human microvascular endothelial cells *in vitro* [164]. In rat small intestine platelet-activating factor promotes mucosal apoptosis via Fas ligand-mediating caspase-9 active pathway [165]. Vitamin D_3_ induces caspase-14 expression and enhances caspase-14 activation in processing organotypic skin cultures [166]. Ceramide stimulates caspase-3, -5, -7, -8, -9 and -14 activities leading to apoptosis in many cells and tissues [166,167,168,169,170,171]. These lipids can be used or developed as anticancer drugs (Figure 4).

### 5.3. Lipid Metabolism and Drug Resistance

In spite of many significant progresses in cancer therapy, cancer is still a major disease that causes more than 8 million deaths, or about 15% of all human deaths around the world every year because most cancer patients eventually develop drug resistance [172]. Drug resistance of cancer cells represents a serious barrier to successful clinic treatment, and inherent drug resistance of cancer cells is caused by multiple mechanisms. The molecular mechanisms of drug resistance can be caused by gene mutations which can enzymatically deactivate the drug, alter the drug-specific binding site, decrease drug permeability and/or increase active efflux (pumping out) of the drugs across plasma membrane, and/or change of the metabolic pathway to yield different non-cytotoxic products. Many of these processes are associated with the alteration of lipid metabolism. Ceramide is a center of sphingolipid metabolism, and more than eleven different enzymes use ceramide as a substrate (ceramidase, ceramide kinase, glycosylceramide synthase, galactosylceramide synthase, and sphingomyelin synthase) or directly convert other molecules to ceramide (dihydroceramide desaturase, sphingomyelinase, ceramide-1-phosphate phosphatase, glucocerebrosidase, galactocerebrosidase, and ceramide synthase) [75,163]. One of the best examples of how changing metabolic pathways may lead cancer cell to drug resistance is that ceramide-generating cancer chemotherapeutic drugs impact the accumulation of ceramide [163] and the increased levels of cellular ceramide drives cancer cell death [73]. Accumulation of cellular ceramide also activates glucosylceramide synthase which converts ceramide to glucosylceramide, thereby reducing ceramide levels in the cells [75]. Glucosylceramide has been demonstrated to stimulate cell growth and DNA synthesis which drive cancer cell resistance to chemotherapy [173]. Other ceramide derivatives such as ceramide-1-phosphate and sphingosine-1-phosphate also regulate cell survival and proliferation pathways, and could lead to drug resistance, as well [161].

## 6. Conclusions

Lipid metabolism is very complex and is regulated by a complex signaling network in the cells. The same lipid molecule, via different signaling pathways or under different conditions, can generate different metabolites. Understanding and defining signaling pathways of lipid metabolism in cancer cells can provide rational targets for therapy, and determining the function of different lipid molecules could develop new anticancer drugs for clinical evaluation. Clearly, better understanding of lipid metabolism in cancer therapy and apoptosis requires further elucidation and investigation to develop new and better cancer treatments for future cancer patients.

## Abbreviations

ABCA: ATP-binding cassette transporter subfamily A1
Aβ: amyloid beta peptides
Apaf: apoptotic protease activating factor
ApoL: apolipoprotein L
ApoE: apolipoprotein E
CHOP: C/EBP-homologous protein
ERK: Extracellular signal-regulated kinase
GPCR: G protein-coupled receptor
HDL: high-density lipoproteins
HMG-CoA: 3-Hydroxy-3-methyl-glutaryl-CoA
JNK: c-jun *N*-terminal kinase
LDL: low-density lipoproteins
MAM: mitochondria associated membrane
MAPK: mitogen-activated protein kinase
miRNA: microRNA
mTOR: mammalian target of rapamycin
PA: phosphatidic acid
PUMA: proteins p53-upregulated modulator of apoptosis
ROS: reactive oxygen species
SRBF: sterol-regulatory binding factor
TNF: tumor necrosis factor
RAIL: TNF-related apoptosis-inducing ligand
